# Trauma Exposures, Resilience Factors, and Mental Health Outcomes in Persons Granted Asylum in the U.S. for Claims Related to Domestic Violence and Persecution by Organized Gangs

**DOI:** 10.1007/s10903-021-01324-2

**Published:** 2021-12-21

**Authors:** Eleanor H. Emery, Mehar Maju, Kate Coursey, Cameron Brandt, Jamie S. Ko, Kathryn Hampton, Adam Richards

**Affiliations:** 1 Center for Health Equity Education & Advocacy, Cambridge Health Alliance, 1493 Cambridge Street, Macht 420, Cambridge, MA 02139 USA; 2 Department of Internal Medicine, Northern Navajo Medical Center, Shiprock, NM USA; 3 Department of Community Health Sciences, UCLA Fielding School of Public Health, Los Angeles, CA USA; 4 Department of Medicine, David Geffen School of Medicine at UCLA, Los Angeles, CA USA; 5 Asylum Program, Physicians for Human Rights, New York, NY USA; 6 Department of Global Health, Milken Institute School of Public Health, The George Washington University, Washington, DC USA

**Keywords:** Asylum, Domestic violence, Persecution by organized gangs, Mental health, Resilience

## Abstract

Survivors of domestic violence (DV) and of violence perpetrated by organized gangs (GV) face barriers to legal protection under U.S. asylum law. We abstracted data from 132 affidavits based on forensic medical evaluations of asylum seekers granted legal protection in the U.S. on the basis of DV and/or GV. We described claimants’ trauma exposures and resilience factors and used multiple logistic regression to quantify associations with Diagnostic and Statistical Manual-5 (DSM-5) diagnoses and improvement in mental health. People seeking asylum based on DV and/or GV have endured multiple types of trauma with significant impacts on their mental health. New experiences of trauma following migration to the U.S. were common and associated with DSM-5 diagnoses. Conversely, resilience factors were associated with improved mental health. Policies that aim to reduce ongoing trauma in the U.S. and to bolster resilience factors may promote asylee mental health and well-being.

## Background

Survivors of domestic violence (DV) and violence perpetrated by organized gangs (GV) face multiple barriers to legal protection under U.S. asylum law. The Refugee Act of 1980 offered protection primarily to individuals with a well-founded fear of persecution by government (State) actors on the basis of race, religion, nationality, or political opinion. Survivors of DV and/or GV often rely on the fifth, less specific category of “membership in a particular social group,” an ambiguous classification with additional legal qualifications. The interpretation of this category varies by Circuit Court jurisdiction and with changing presidential administrations, exposing applicants to legal vulnerabilities. Most survivors of DV and/or GV face persecution by non-State actors and therefore must demonstrate that the government is unable or unwilling to control their persecutors, which increases their evidentiary burden. In June of 2018, Attorney General Sessions explicitly asserted that claims “pertaining to domestic violence or gang violence perpetrated by non-governmental actors will not qualify for asylum” [1]. Although a Federal Judge later blocked the application of this case certification to the credible fear process [2], uncertainties surrounding DV and/or GV asylum claims persist. In 2021, the Biden administration initiated a review of whether the U.S. provides protection for those fleeing DV and/or GV in a manner consistent with international standards [3], which was ongoing at the time of writing.

Prior studies have shown that many forced migrants, including asylum seekers, experience multiple episodes of physical, sexual and psychological abuse in their home countries prior to fleeing [4–6] and that these traumas have lasting adverse impacts on their physical and mental health and quality of life [4–11]. An emerging body of evidence suggests that the post-migration experience also shapes the mental health prognosis of these populations. Whereas ongoing trauma following migration may increase one’s risk for adverse mental health outcomes [7, 12–17] individual and community resilience factors can decrease the burden of mental illness [18]. Most studies on resilience are limited by their reliance on patient-completed questionnaires to evaluate mental health outcomes rather than diagnostic evaluations by licensed clinicians, and studies have not specifically explored the experiences and mental health outcomes among DV and/or GV asylum seekers.

This mixed-methods study uses data contained in affidavits from forensic medical evaluations (FMEs) of individuals granted asylum on grounds of DV and/or GV to accomplish the following aims: first, to describe patterns of trauma, impunity for perpetrators, and resilience within these populations, and second, to explore how trauma and resilience impact mental health outcomes in the post-migration period.

## Methods

### Applicants

We abstracted data from 132 affidavits written by clinicians in the national Physicians for Human Rights (PHR) Asylum Network based on FMEs of asylum seekers of any age who were successful in obtaining asylum or another form of humanitarian immigration status on the basis of DV, GV, or a combination of the two in their home countries. We only included affidavits for applicants who were successful in order to characterize the trauma exposures and health outcomes of persons whom the legal system determined met the criteria for protection. For the purposes of this study, DV was defined as acts of abuse in the social context of domestic settings, including intimate partner violence and violence perpetrated by a family member or a resident in the home, e.g., a parent or aunt [19]. Among 1,944 de-identified affidavits in the PHR database from FMEs conducted between 1996 and 2019, PHR identified 149 that were potentially eligible. Seventeen were subsequently excluded, including fifteen where the DV and/or GV occurred exclusively in the U.S. and two where the client was evaluated as part of a family member’s case but had not personally experienced violence. All FMEs were conducted in the U.S.

### Data Collection

Our multidisciplinary research team, composed of PHR staff, physicians and postgraduate students, developed a coding tool based on one previously used to abstract quantitative and qualitative data from medical affidavits [20]. Diagnostic criteria from the Diagnostic and Statistical Manual of Mental Disorders-5 (DSM-5) were used to define mental health diagnoses. Resilience was assessed using questions on factors determined in prior studies to positively affect mental health and well-being in displaced populations [18].

A draft of the coding tool was reviewed by independent experts in immigration law, FMEs, and the clinical care of asylum seekers. The final tool consisted of 54 categorical and free-response items that were inputted into the survey platform Qualtrics®. Authors AR, EE, and MM trained a group of medical and graduate students on systematic data abstraction methods and reviewed the accuracy of abstracted data from the first coded affidavits. We assessed all abstracted data with range and consistency checks and reviewed original affidavits for missing values of key variables.

### Measures

We analyzed the abstracted data for the following: client demographics, trauma exposures, perpetrator characteristics, resilience factors, and physical and mental health outcomes including DSM-5 diagnoses as determined by the clinician who authored the affidavit. Data pertaining to mental health outcomes were abstracted from affidavits resultant of forensic mental health evaluations (n = 101). We coded improved mental health when an affidavit made explicit mention of improvement in the client’s mental health or well-being at the time of evaluation relative to their historical reporting of psychological symptoms. Resilience factors were categorized as protective and/or mitigating aspects of a client’s life including family and social support, religion and collective identity, work and school, access to mental and other clinical health services, and individual traits.

### Analysis

We used multiple logistic regression models to quantify associations between client trauma exposures and resilience factors and each of two primary mental health outcomes: any DSM-5 diagnosis and improved mental health symptoms. The adjusted odds ratios provide estimates of the associations between trauma and resilience factors and adverse (DSM-5 diagnosis) or salutary (improved symptoms) mental health outcomes, adjusted for client characteristics.

There were 35 free-text items available for qualitative analysis. Items with over 80 responses were assigned to pairs of study authors who conducted independent thematic analyses and discussed discrepancies until consensus was achieved.

The Institutional Review Board of the University of Los Angeles determined this study of de-identified records to be exempt from ethical review.

The dataset generated and analyzed during this study is available from the corresponding author on reasonable request.

## Results

### Demographics and Sample Characteristics

The majority of applicants identified as female (n = 101, 77%), were under the age of 45 (n = 116, 88%), and were evaluated between 2009 and 2015 (n = 85, 64%; Table 1). The proportion of applicants from Northern Triangle countries was 55% overall, and increased over time from 23% (1999–2008) to 64% (2009–2019, p < 0.001, data not shown). There were no significant differences between DV and GV applications with the exception that the former were more likely to be female (80% versus 61%, p = 0.01, data not shown). A minority of clients (n = 13, 9.8%) were applying on the grounds of both DV and GV. Most the applicants in this sample (92%) were applying solely on the grounds of DV and/or GV; their applications did not identify any additional grounds for asylum (i.e. race, religion, nationality, political affiliation or membership in another particular social group). The majority of the affidavits featured mental health evaluations (n = 101, 77%); 14% (n = 19) included both a mental health and a physical evaluation. Three subjects identified as gay men and two as lesbian women; the remainder identified as heterosexual and none identified as transgender.

**Table 1:** Characteristics and trauma exposures of 132 individuals seeking asylum in the U.S. on the basis of domestic violence and/or violence by organized gangs 1999–2019

	N	%
*Age*		
< 18	17	13
18–24	31	23
25–34	41	31
35–44	29	20
≥ 45	16	12
*Gender*		
Female	101	77
*Country of origin*		
Northern Triangle^a^	73	55
Other Latin America (includes Mexico)	15	11
Sub-Saharan Africa	33	25
Other	11	8
*Year of forensic evaluation*		
1999–2008	30	23
2009–2015	84	64
2016–2019	17	13
*Basis of asylum claim*^b^		
Domestic violence	107	81
Gang violence	38	29
Both	13	10
*Evaluation type*		
Mental health	101	77
Physical	50	38
Gynecological	7	5
*Trauma location*		
Home country	132	100
In transit	15	11
Since arrival in the U.S	61	46
*Trauma categories experienced*^cd^		
Physical violence	97	73
Sexual violence	90	68
Other forms of abuse	116	88
Indirect trauma/abuse	92	70
Targeted economic marginalization	54	41
*Number of trauma categories experienced*^d^		
1	6	5
2	23	18
3	35	27
4	48	36
5	20	15
*Total number of trauma types experienced*^de^		
1 to 5	46	35
6 to 10	75	57
> 10	11	8
*Experience of Impunity*^f^		
Reported impunity	30	23

^a^El Salvador, Guatemala and Honduras

^b^Compared to survivors of gang violence, domestic violence survivors were more likely to be women (80% vs. 61%, p = 0.01) and less likely to experience trauma during transit (8% vs. 18%, p = 0.03); all other comparisons not significant (p > 0.05)

^c^See Fig. 1 for description of the five trauma categories

^d^Trauma categories and types of trauma experienced refers to trauma in the home country

^e^Twenty six (26) trauma types were coded. See Fig. 1 for complete list

^f^Experience of impunity was coded as present if an individual 1) sought assistance from authorities and was met with an inappropriate response (no response or unprofessional conduct/further persecution) or 2) described a fear of retribution or general community perception/knowledge of impunity/lack of accountability

### Trauma Exposures

**Fig. 1:**
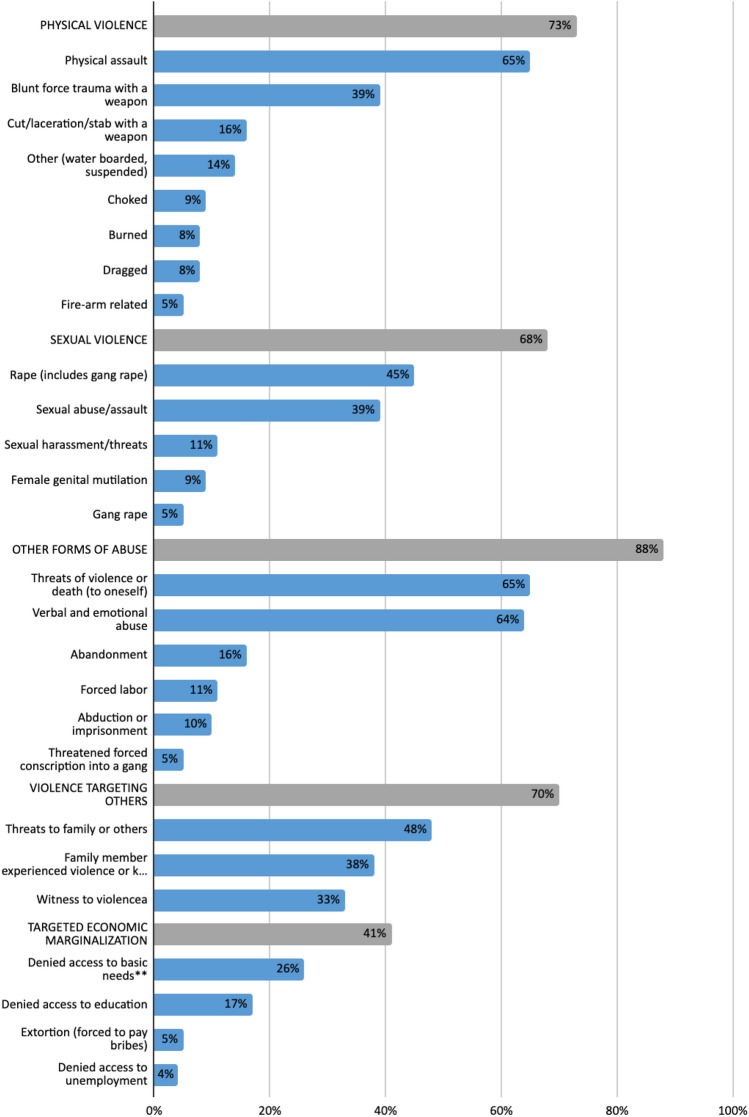
Trauma exposures reported by 132 individuals seeking asylum in the U.S. on the basis of domestic violence and/or violence by organized gangs. Figure 1 displays the percentage of clients whose affidavits mentioned each of 26 types of trauma (speckled bars) grouped into five trauma categories (solid bars, labeled in caps). ^a^Physical violence included being subjected to physical assault; blunt force trauma with a weapon; cut, stabbed, or lacerated with a weapon; shot with a firearm; burned; dragged; choked; kidnapped/detained; or other forms of physical torture (e.g. water boarded or suspended from the ceiling). ^b^Sexual violence included sexual harassment/threats; sexual abuse/assault; rape; gang rape; or female genital mutilation. ^c^Other abuse included forced labor; verbal or emotional abuse; abandonment; abduction or imprisonment; threats of forced conscription into gangs; or threats of violence or death to oneself. ^d^Violence targeting other included threats of violence against family members or others; having a family member who experienced violence or was killed; or witnessing violence against others (e.g. witnessing, aggravated assault, rape or murder). ^e^Witness to violence such as aggravated assault, rape, murder. ^f^Targeted economic marginalization included being deprived of access to education; being deprived of access to employment (e.g. being fired unfairly); extortion (e.g. being forced to pay bribes); or being deprived of access to basic needs (e.g. food, clothing, or shelter) or other forms of neglect. ^g^Denied access to basic needs such as food, clothing or shelter

Applicants reported a wide range of trauma exposures (Fig. 1). These trauma exposures were grouped into five categories: physical violence, sexual violence, other abuse including verbal and emotional, violence targeting others including threats to or violence against loved ones, and targeted economic marginalization including extortion. We found that nearly all (95%) applicants had experienced multiple categories of trauma, and 78% had experienced three or more categories. Sexual violence was reported by 68% of applicants overall, and was more frequently reported by female applicants (85% versus 13% in men, p < 0.01, data not shown). There were no significant differences in the types of trauma categories reported by DV and GV applicants.

### Perpetrators

A substantial proportion of applicants reported multiple perpetrators of both physical (28%) and sexual (22%) violence. Of the seven coded categories of perpetrators, intimate partners were the most commonly identified perpetrators of physical (57%) and sexual violence (69%) overall. Survivors of GV reported also experiencing one or more episodes of violence by intimate partners, who in some but not all cases were gang members, 39% of the time, whereas survivors of DV reported experiencing violence by intimate partners (as opposed to other household members) 64% of the time (p < 0.01, data not shown). In GV claims, gang members themselves were rarely identified as perpetrators of sexual violence (1/21 = 5%) specifically; intimate partners (48%) and extended family members (29%) were more frequently implicated.

### Impunity and Failed Relocation Attempts

Affidavits reported that clients sought assistance from an authority figure (police, military, or government official or civil society organization) in their home country in 25% of cases. In 81% of these cases there was either no response or the report resulted in unprofessional conduct or further persecution. In 11% of affidavits, the client gave an explicit reason why they did not seek assistance from authorities, including prior experience with impunity, fear of retribution, or community perceptions regarding the permissibility of their abuse. Over a third of asylum seekers (37%) attempted to relocate to find safety within their home country or to another country in the region prior to migrating to the U.S., including 22 clients who attempted to relocate more than once.

### Trauma After Arrival in the U.S.

Almost half (46%) of applicants reported experiencing abuse after arrival in the U.S. (Table 1). This abuse included ongoing threats to themselves and/or their loved ones by perpetrators in their home country and new forms of abuse in the U.S., including harassment or assault in the workplace or abuse by a new intimate partner in the U.S.

### Mental Health Outcomes

Of the 101 applicants in this sample that received a mental health evaluation, 79% were determined by the clinician who authored the affidavit to have met criteria for one or more DSM-5 diagnoses (Table 2). The most common DSM-5 diagnosis was post-traumatic stress disorder (PTSD, 68% of clients), followed by major depressive disorder (38%). Suicidality also was common, with 32% of clients reporting any history of suicidal ideation or attempt and 13% reporting active suicidality at the time of the evaluation, though it was not specified whether this was related to their experience of DV and/or GV. Among affidavits with a mental health evaluation, 42% described an improvement in the applicant’s symptoms or well-being by the time of the evaluation. For the majority of these applicants, the improvement was specified to have occurred since migration to the U.S.

**Table 2:** Mental health diagnoses, symptom improvement and resilience factors among 101 asylum seekers with forensic mental health evaluations

	N	%
Currently meets DSM-5 diagnostic criteria	81	79
Post-traumatic stress disorder	69	68
Major depressive disorder	38	38
Generalized anxiety disorder	10	10
Other DSM-5 diagnosis^a^	9	9
(Likely) met diagnostic criteria in the past	8	8
Major depressive disorder	4	4
Post-traumatic stress disorder	3	3
Other DSM-5 diagnosis	1	1
*Suicidality*		
Suicidal ideation or attempt (ever)^b^	32	32
Active suicidal ideation	13	13
Suicide attempt	15	15
*Improved mental health*		
Improved mental health symptoms^c^	42	42
Suicidality resolved	19	19
*Resilience factors*		
Family and social support	43	43
Religion & collective identity	23	23
Work and school	11	11
Mental and other clinical health services	5	5
Individual/internal locus of resilience	2	2
*Number of resilience factors coded*		
None	43	43
One	35	35
Two or more	23	23

*DSM-5* Diagnostic and Statistical Manual of Mental Disorders-5

^a^Other DSM diagnoses were Persistent Depressive Disorder/Dysthymia (n = 5), and Adjustment Disorder, Somatization Disorder, Other Depressive Disorder, Other Specified Trauma- and Stressor-Related Disorder (n = 1 each)

^b^Frequency of all mental health outcomes and resilience factors was similar (p > 0.05) for survivors of gang violence (GV) and domestic violence (DV) with the exception of suicidal ideation or attempt, which was more common among survivors of GV than DV (50% vs. 26%, p = 0.01)

^c^Improved mental health symptoms were explicitly described in the affidavit; also includes one client who likely met criteria in the past for post-traumatic stress disorder but no longer met criteria at the time of the evaluation. Comparator group is no change or worsening of symptoms

### Associations between Mental Health Outcomes and Exposure to Ongoing Trauma and Resilience Factors

**Fig. 2:**
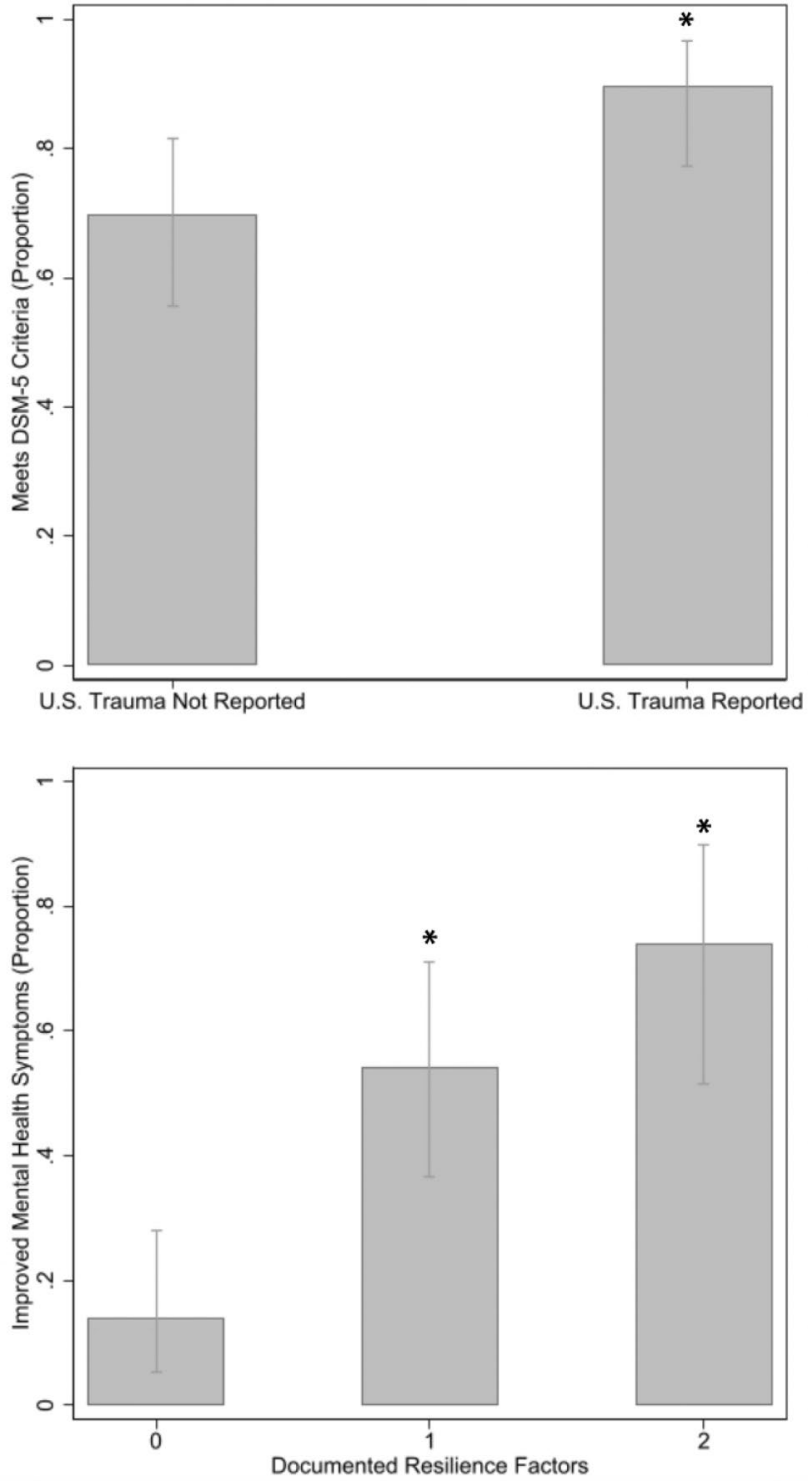
Association of mental health outcomes with trauma since arrival in the U.S. and documented resilience factors. The upper panel shows that Diagnostic and Statistical Manual of Mental Disorders-5 (DSM-5) criteria were met for a higher proportion of the sample that experienced trauma after arrival in the U.S. (n = 61) compared with those that did not (n = 71; p = 0.01). The lower panel shows that the proportion of the sample that experienced an improvement in mental health symptoms was higher for clients with one (n = 35), or with two or more resilience factors (n = 23) noted in the affidavit (p < 0.01 for both categories), compared to clients without noted resilience factors (n = 43). See text for a description of resilience factors

Asylum seekers who experienced trauma after arrival in the U.S. were more likely to meet criteria for a DSM-5 diagnosis compared to those who did not (90% vs. 70%, p = 0.01; Fig. 2), and this association remained significant in the multivariable model (adjusted OR 4.5, p = 0.03; Table 3).

**Table 3:** Adjusted associations^a^ of trauma exposures and resilience factors to DSM-5 diagnosis or improved mental health symptoms among 101 asylum seekers with forensic mental health evaluations

	Currently meets criteria for one or more DSM-5 diagnosis				Currently meets criteria for improved mental health symptoms			
	%	Adjusted OR^bc^	95% CL		%	Adjusted OR^bd^	95% CL	
*Number of trauma categories experienced*^ef^								
1–2	90%	not included in the final model			50%	–	–	–
3–4	78%				30%	**0.5**	**0.2**	**3.6**
5	72%				72%	**2.6**	**0.2**	**27.9**
*Trauma since arrival in the U.S.*								
No trauma in U.S.	70%	–	–	–	45%	not included in the final model		
Trauma since U.S. arrival	90%	**4.5**	**1.1**	**17.4**	39%			
*Documented resilience factors*								
0	86%	–	–	–	14%	–	–	–
1	80%	**0.5**	**0.1**	**2.3**	54%	**7.9**	**2.4**	**25.7**
2 or more	65%	**0.5**	**0.1**	**2.3**	74%	**15.0**	**3.9**	**58.4**

*DSM-5* Diagnostic and Statistical Manual of Mental Disorders-5, *OR* odds ratio, *CL* confidence limit, significant associations (p < 0.05) are shown in bold

^a^Candidate variables evaluated for possible inclusion in adjusted analyses included country of origin, year of forensic evaluation, basis for asylum claim, trauma categories (evaluated independently and as a cumulative number of categories), trauma since arrival in the U.S. and number of documented resilience factors

^b^Adjusted models included age, gender and variables with significant associations in the crude model (p < 0.1)

^c^Adjusted model for the outcome of any DSM-5 diagnosis included age, gender, physical violence, indirect trauma, targeted economic marginalization and trauma since arrival in the U.S

^d^Adjusted model for the outcome of improved mental health symptoms included age, gender, and total number of trauma categories and documented resilience factors

^e^See Fig. 1 for description of the five trauma categories

^f^Trauma categories experienced refers to trauma in the home country

Over half (58%) of affidavits with a mental health evaluation included information on resilience factors, protective and/or mitigating aspects of a client’s life that helped promote the client’s recovery from trauma (Table 2). The most common of these were family and social support (43%) and religion and collective identity (23%), including the opportunity for community engagement by attending religious services. Improvement in mental health symptoms demonstrated a significant, dose–response relationship with the number of reported resilience factors, from 14% of clients who reported no resilience factors to 74% of clients who reported two or more (adjusted OR 15.0, p =  < 0.01; Table 3 and Fig. 2).

## Discussion

People seeking asylum in the U.S. on the basis of DV and/or GV in their home countries have endured multiple types of trauma, including physical and sexual violence, at the hands of multiple perpetrators, with significant impact on their mental health. Prior studies of forced migrants have documented frequent exposures to multiple sources of severe trauma [4–6, 11], and many studies have found high rates of mental illness [4, 9, 15, 21], though rates can vary dramatically between different groups of forced migrants and when different study methodologies are utilized [8, 22]. We extend this evidence of significant trauma and mental health burden to individuals seeking asylum in the U.S. on the grounds of DV and/or GV.

Almost half of the clients in our sample experienced trauma after arrival in the U.S., and this was associated with a higher risk of DSM-5 diagnosis. Post-migration trauma included ongoing threats to themselves and/or their loved ones by perpetrators in their home country, which speaks to the well-founded fear of future persecution by these individuals if applicants were to return to their home countries. Others reported new experiences of trauma from U.S-based perpetrators, including harassment or assault in the workplace or abuse by a new intimate partner. This is a testament to the ongoing vulnerability of these populations, particularly during the period when the applicant does not yet have legal status in the U.S. and may fear that reporting abuse would put them at risk of deportation. Conversely, resilience factors, including family and social support and religion and collective identity, were associated with improved mental health symptoms. Our findings contribute to a growing body of evidence that the post-migration experience impacts prognosis [7, 12–14, 16–18].

Our results also highlight the culture of impunity that is present in applicants’ countries of origin and legitimizes their need to flee. Many clients in this sample either attempted to seek assistance from the State without success or specifically stated that they did not seek assistance because a culture of impunity made reporting futile and could place them or their loved ones at risk of retaliation [9]. This lack of accountability is likely underrepresented in our sample because information about the State’s response is often not included in the medical affidavit. Nevertheless, the pervasiveness of this culture of impunity calls into question the legal requirement that one proves “complete helplessness” on the part of the State to prevent persecution by non-governmental actors [23]**,** such as intimate partners or gangs, as many who experience this persecution will not request assistance from the State when they know this is futile or may result in retaliation.

Strengths of our study include its focus on individuals seeking asylum on grounds related to DV and/or GV. Previous studies of post-migration factors and mental health in forced migrants are not specific to asylum seekers with DV and/or GV claims, an important group in the context of the increasing number of migrants fleeing endemic violence in Central America [24–28]. We collected data from detailed medical affidavits written by licensed clinicians rather than self-reported surveys, strengthening the validity of mental health findings and providing a narrative context to categorize traumatic exposures and resilience factors.

This study had several limitations. First, the sample was limited to clients in the PHR database who were granted legal protection and thus the findings are not necessarily generalizable to all DV and/or GV asylum seekers, particularly those who lacked legal representation and whose claims were denied. Sample characteristics also did not account for racial or ethnic identification and findings may not represent the experience of all racial or ethnic groups. The small number of LGBTQIA + clients in our sample limits our ability to generalize findings to LGBTQIA + applicants. Finally, our study was a retrospective review of affidavits from FMEs conducted to assist adjudication of asylum claims; they were not designed to evaluate the associations we explored. Information in the affidavits may therefore be influenced by the training and experience of the clinicians and is subject to ascertainment bias.

Our findings underscore the need for policy changes to help bend the mental health trajectory of asylees away from persistent suffering and towards recovery. Legal and medical evaluations of asylees often focus on abuse in the home country and may be missing the opportunity to identify and intervene upon current trauma occurring in the U.S. Policies should aim to protect asylum seekers from trauma in the U.S. by making screening for current DV commonplace in legal and medical asylum evaluations and increasing the availability of DV services for applicants. Applicants must be informed of their rights under U.S. law and be encouraged to report abuse in the U.S. Policy changes must also specifically discourage collaboration between immigration and law enforcement, which can make those without legal status hesitant to report crimes in the U.S.

Policies that promote resilience factors should also be prioritized, such as timely family reunification and alternatives to detention, which precludes connections with social networks and community resources. Temporary work visas and shorter waiting periods for asylum hearings would enhance asylum seekers’ access to the material and mental health benefits of gainful employment. Connections to medical and in particular mental healthcare, as well as to educational opportunities, religious institutions and other sources of community, would further promote recovery. Other countries have enacted policies designed to promote the health and social inclusion of resettled refugees and asylees and studies are needed to assess the impacts of these policies [29–31].

In summary, this study provides a detailed assessment of the multiple and severe traumas experienced by DV and/or GV asylum seekers and how their post-migration experiences can exacerbate or begin to ameliorate their high burden of mental suffering. Experiences of trauma following migration to the U.S. were common and associated with DSM-5 diagnoses; conversely, resilience factors were associated with improved mental health symptoms. Findings can be used to inform immigration policy priorities, such as the importance of screening asylum applicants for DV and other forms of current trauma in the U.S., and connecting applicants with sources of resilience that they identify, including family and religious community.

## References

[1] Chang Newell J. Federal Judge Blocks Trump’s Policy Gutting Asylum for People Fleeing Domestic and Gang Violence [Internet]. American Civil Liberties Union https://www.aclu.org/blog/immigrants-rights/federal-judge-blocks-trumps-policy-gutting-asylum-people-fleeing-domestic-and (2018). Accessed 21 Mar 2021.

[2] Grace v. Whitaker, 344 F. Supp. 3d 96 (D.D.C. 2018). https://www.aclu.org/sites/default/files/field_document/opinion_-_grace_v._whitaker.pdf (2021). Accessed 22 Mar 2021.

[3] Biden JR. Executive Order 14010: Creating a Comprehensive Regional Framework to Address the Causes of Migration, to Manage Migration Throughout North and Central America, and to Provide Safe and Orderly Processing of Asylum Seekers at the United States Border. United States. Office of the Federal Register. https://www.hsdl.org/?view&did=849584 (2021). Accessed 21 Mar 2021.

[4] Steel Z, Chey T, Silove D, Marnane C, Bryant RA, van Ommeren M (2009). Association of torture and other potentially traumatic events with mental health outcomes among populations exposed to mass conflict and displacement: a systematic review and meta-analysis. JAMA.

[5] Aguirre NG, Milewski AR, Shin J, Ottenheimer D (2020). Gender-based violence experienced by women seeking asylum in the United State: A lifetime of multiple traumas inflicted by multiple perpetrators. J Forensic Leg Med.

[6] Cunningham M, Cunningham JD (1997). Patterns of symptomatology and patterns of torture and trauma experiences in resettled refugees. Aust N Z J Psychiatry.

[7] van der Boor CF, Amos R, Nevitt S, Dowrick C, White RG (2020). Systematic review of factors associated with quality of life of asylum seekers and refugees in high-income countries. Confl Health.

[8] Due C, Green E, Ziersch A (2020). Psychological trauma and access to primary healthcare for people from refugee and asylum-seeker backgrounds: a mixed methods systematic review. Int J Ment Health Syst.

[9] Keller A, Joscelyne A, Granski M, Rosenfeld B (2017). Pre-migration trauma exposure and mental health functioning among central american migrants arriving at the US border. PLoS ONE.

[10] Heslehurst N, Brown H, Pemu A, Coleman H, Rankin J (2018). Perinatal health outcomes and care among asylum seekers and refugees: a systematic review of systematic reviews. BMC Med.

[11] Knipscheer JW, Sleijpen M, Mooren T, ter Heide FJJ, van der Aa N (2015). Trauma exposure and refugee status as predictors of mental health outcomes in treatment-seeking refugees. BJPsych Bull.

[12] Li SSY, Liddell BJ, Nickerson A (2016). The relationship between post-migration stress and psychological disorders in refugees and asylum seekers. Curr Psychiatry Rep.

[13] Yun S, Ahmed SR, Hauson AO, Al-Delaimy WK (2021). The Relationship Between Acculturative Stress and Postmigration Mental Health in Iraqi Refugee Women Resettled in San Diego. California. Community Ment Health J.

[14] Porter M, Haslam N (2005). Predisplacement and postdisplacement factors associated with mental health of refugees and internally displaced persons: a meta-analysis. JAMA.

[15] Henkelmann J-R, de Best S, Deckers C, Jensen K, Shahab M, Elzinga B, Molendijk M (2020). Anxiety, depression and post-traumatic stress disorder in refugees resettling in high-income countries: systematic review and meta-analysis. BJPsych Open.

[16] Kirmayer LJ, Narasiah L, Munoz M, Rashid M, Ryder AG, Guzder J, Hassan G, Rousseau C, Pottie K (2011). Canadian Collaboration for Immigrant and Refugee Health (CCIRH): Common mental health problems in immigrants and refugees: general approach in primary care. CMAJ.

[17] Asgary R, Segar N (2011). Barriers to health care access among refugee asylum seekers. J Health Care Poor Underserved.

[18] Siriwardhana C, Ali SS, Roberts B, Stewart R (2014). A systematic review of resilience and mental health outcomes of conflict-driven adult forced migrants. Confl Health.

[19] Dynamics of Domestic Violence [Internet]. Domestic Violence Coordinating Council. https://dvcc.delaware.gov/background-purpose/dynamics-domestic-abuse/ (2011)

[20] Aguirre NG, Milewski AR, Shin J, Ottenheimer D (2020). A coding tool and abuse data for female asylum seekers. Data Brief.

[21] Fazel M, Wheeler J, Danesh J (2005). Prevalence of serious mental disorder in 7000 refugees resettled in western countries: a systematic review. Lancet.

[22] Blackmore R, Boyle JA, Fazel M, Ranasinha S, Gray KM, Fitzgerald G, Misso M, Gibson-Helm M (2020). The prevalence of mental illness in refugees and asylum seekers: A systematic review and meta-analysis. PLoS Med.

[23] Matter of A-B-, Respondent. 28 I&N Dec. 199 (A.G. 2021). https://www.justice.gov/eoir/page/file/1354636/download (2021). Accessed 22 Mar 2021.

[24] Baugh R. Refugees and Asylees: 2019 [Internet]. Department of Homeland Security Office of Immigration Statistics. https://www.dhs.gov/sites/default/files/publications/immigration-statistics/yearbook/2019/refugee_and_asylee_2019.pdf (2020). Accessed 22 Mar 2021.

[25] Wilson JH, Bruno A, Elsea JK, Kandel WA, Kapp L, Margesson R, Meyer PJ, Ribando Seelke C, Singer A, Taft-Morales M. Recent Migration to the United States from Central America: Frequently Asked Questions [Internet]. Congressional Research Service. https://fas.org/sgp/crs/row/R45489.pdf (2019). Accessed 22 Mar 2021.

[26] Cardoletti-Carroll C, Farmer A, Vélez LE. Women on the Run: First-Hand Accounts of Refugees Fleeing El Salvador, Guatemala, Honduras, and Mexico [Internet]. United Nations High Commissioner for Refugees. https://www.unhcr.org/56fc31864.html (2015). Accessed 22 Mar 2021.

[27] Soszynska M. Forced to Flee Central America’s Northern Triangle: A Neglected Humanitarian Crisis [Internet]. Medecins Sans Frontieres. https://www.msf.org/sites/msf.org/files/msf_forced-to-flee-central-americas-northern-triangle_e.pdf (2017). Accessed 22 Mar 2021.

[28] World Bank. Crime and Violence in Central America: A Development Challenge - Main Report [Internet]. World Bank. https://openknowledge.worldbank.org/handle/10986/2744 (2011). Accessed 22 Mar 2021.

[29] Repository of promising practices—Employment, Social Affairs & Inclusion [Internet]. European Commission. https://ec.europa.eu/social/main.jsp?langId=en&catId=1208. Accessed 21 Mar 2021.

[30] Papadopoulou A, Treviranus B, Moritz T, Fandrich CM. Comparative study on the best practices for the integration of resettled refugees in the EU Member States [Internet]. European Union - Policy Department C: Citizen’s Rights and Constitutional Affairs. http://www.resettlement.eu/sites/icmc.tttp.eu/files/EP%20study.pdf (2013). Accessed 21 Mar 2021.

[31] Fratzke S. Weathering Crisis, Forging Ahead: Swedish Asylum and Integration Policy. Migration Policy Institute. https://www.migrationpolicy.org/sites/default/files/publications/TCM-Asylum-Sweden-FINAL.pdf (2017)

